# The Double Face of Exosome-Carried MicroRNAs in Cancer Immunomodulation

**DOI:** 10.3390/ijms19041183

**Published:** 2018-04-13

**Authors:** Romina Alfonsi, Ludovica Grassi, Michele Signore, Désirée Bonci

**Affiliations:** 1 Institute of General Pathology, Università Cattolica and Policlinico Gemelli, 00168 Rome, Italy; 2 Department of Oncology and Molecular Medicine, Istituto Superiore di Sanità, 00161 Rome, Italy; ludovica.grassi89@yahoo.it (L.G.); desiree.bonci@iss.it (D.B.); 3 Department of Internal Medicine and Medical Specialties, “La Sapienza” University, 00161 Rome, Italy; 4 Regina Elena National Cancer Institute, 00144 Rome, Italy; 5 RPPA Unit, Proteomics Area, Core Facilties, Istituto Superiore di Sanità, 00162 Rome, Italy; michele.signore@iss.it

**Keywords:** tumor-derived exosomes (TEXs), microRNAs, immune system, cancer

## Abstract

In recent years many articles have underlined the key role of nanovesicles, i.e., exosomes, as information carriers among biological systems including cancer. Tumor-derived exosomes (TEXs) are key players in the dynamic crosstalk between cancer cells and the microenvironment while promote immune system control evasion. In fact, tumors are undoubtedly capable of silencing the immune response through multiple mechanisms, including the release of exosomes. TEXs have been shown to boost tumor growth and promote progression and metastatic spreading via suppression or stimulation of the immune response towards cancer cells. The advantage of immunotherapeutic treatment alone over combining immuno- and conventional therapy is currently debated. Understanding the role of tumor exosome-cargo is of crucial importance for our full comprehension of neoplastic immonosuppression and for the construction of novel therapies and vaccines based on (nano-) vesicles. Furthermore, to devise new anti-cancer approaches, diverse groups investigated the possibility of engineering TEXs by conditioning cancer cells’ own cargo. In this review, we summarize the state of art of TEX-based immunomodulation with a particular focus on the molecular function of non-coding family genes, microRNAs. Finally, we will report on recent efforts in the study of potential applications of engineered exosomes in cancer immunotherapy.

## 1. Introduction

### 1.1. Exosome Definition

Cells release different types of vesicles called extracellular vesicles (EVs) in body fluids. EVs are classified according to their size, biogenesis, and secretion modalities [1,2]. Among EVs, exosomes are nanovesicles of endosomal origin with a diameter ranging from 30 to 100 nm. The term exosomes was coined in 1981 by Trams et al. and referred to “exfoliated membrane vesicles with 5′ nucleotidase activity” [3]. These nanovesicles can shuttle several biomolecules such as carbohydrates, lipids, proteins, and nucleic acids and play a fundamental role in cell communications (Available online: www.exocarta.org/). Exosomes are released from all cell types including tumor cells, which aberrantly produce high levels of vesicles [4]. Although the tetraspanin proteins CD9, CD63, CD81, and CD82 are considered as distinguishing hallmarks of exosomes [5], several lines of evidence indicate that tumor-derived exosomes (TEXs) retain specific surface markers [6] and molecular cargo [7,8]. These observations are at least in part a direct consequence of the molecular settings that exosomes inherit from parental cells. Undoubtedly, it has been shown that exosomes are enriched for specific cellular components [9,10,11,12].

### 1.2. Exosomes Biogenesis

Exosomes originate from the endosomal compartment and encompass two phases [1,13]. The first step involves endocytosis of the plasma membrane leading to the formation of early endosomes. During this step, clathrin-coated, intraluminal vesicles are generated within the early endosome. The second phase of exosome biogenesis occurs through a ceramide-triggered process with inward budding from the outer membrane of endosomes. During exosomes formation, cytosol elements like RNA and proteins are trapped inside lumen. The “Endosomal Sorting Complexes Required for Transport” (ESCRT) in cooperation with accessory proteins such as ALIX (ALG-2 interacting protein X) and Tumor-Susceptibility Gene 101 (TSG101) play an important role in exosome and cargo biogenesis [14]. In particular, ESCRT is responsible for the internalization of ubiquitinated proteins into microvesicular bodies (MVB) [15], and it is believed to cooperate in sorting protein cargo into exosomes. An alternative but not a well characterized mechanism called the ESCRT-independent pathway has also been documented [16]. Reportedly, such a process involves lipids such as sphingosine-1-phosphate and ceramide, as well as tetraspanin-enriched microdomains and sphingomyelinase. However, the rules of cargo shuttling into nanovesicles remain unclear. The trafficking and release of exosomes from the cell membrane have been attributed to being part of the Rab GTPase family, e.g., Rab GTPases 27a and 27b [17,18]. It is of note that their secretion can also be triggered by hypoxia, a common condition in tumors [19].

### 1.3. Exosome Cargo

Exosomal cargo represents an intriguing novel focus of investigation that is likely to improve our knowledge of the molecular mechanisms behind exosome formation and secretion. Moreover, it represents a powerful source of information about parental cancer, which is of critical importance when the tumor tissue is not available for monitoring therapeutic responses [20]. Exosomes shuttle a plethora of molecules including proteins, lipids, and nucleic acids and may therefore offer a comprehensive “portrait” of the aberrant cancer traits. Since RNAs represent the most abundant and variegated compartment, they represent a great and abundant field of study for biomarkers and molecular mechanism discovery. Exosomes transport coding (mRNA), as well as all sorts of non-coding RNAs, i.e., long non-coding RNA, pseudogene RNA, siRNA (short interfering RNA), piRNA (Piwi-interacting RNA), viral RNA, circular RNA, and microRNA [21,22,23]. Since microRNA gene family (miRs, miRNAs) has been largely studied in cancer [24,25], here we focus our attention on the microRNA exosomal cargo in tumors. In particular, we discuss how cancer-derived microRNAs conveyed by exosomes influence the immune system.

MicroRNAs are small non-coding RNAs usually 19–24 nucleotides in length and sophisticated regulators of coding RNAs. They negatively regulate mRNAs at a post-transcriptional level by binding the candidate targets in specific regions, “binding sites”, causing inhibition of protein translation. Each microRNA acts on multiple mRNA targets, while any mRNA may be blocked by different microRNAs. This intricate network is devoted to maintaining a fine tuning of several biological functions. Since cancers showed miRNA profile aberrant expression, their alteration assumes a key function in tumor progression. To further complicate the scenario, recent studies have implicated tumor exosomal microRNA cargo in intercellular communication and in empowering neoplastic aggressiveness [23,26,27].

Although exosomal microRNA cargo mostly recapitulates parental cells, diverse lines of evidence reported that microRNA are selectively incorporated into exosomes [1,28,29]. Trapping of microRNAs inside exosomes appears to be a non-random process, and it is dictated by specific mechanisms [27,28,29,30,31]. In fact, specific microRNA sequences have been reported to facilitate their uploading into exosomes through specific protein complexes. In addition, it has been hypothesized that exosomal assortment of microRNAs may be negatively correlated with microRNA target abundance. This seems to be correlated with microRNA direct sequestering by mRNA targets, which act like sponges and prevent microRNA loading [26,27]. A further microRNA selection mechanism involving the neural sphingomyelinase 2 (nSMase2) has also been described [32]. In particular, inhibition of nSMase2 has been shown to be reverted by transferring microRNAs from cancer to endothelial cells. These data suggest that nSMase2 may play an important role in microRNA sorting [32].

Similarly, another group reported that exosomes released by T cells are enriched in microRNAs, and that such miRNAs share motives composed by four nucleotides, called EXOmotives, which are putatively responsible for the specific sorting of microRNAs into nanovesicles. In this regard, EXOmotives appear to mediate the interaction of miRNAs with the RNA binding protein, hnRNPA2B1 (heterogeneous ribonucleoprotein A2B1), ultimately leading to miRNA loading into exosomes [29,33,34]. Furthermore, a recent paper showed that Ago2, a member of the RNA-Induced Silencing Complex (RISC), may be involved in the exosomal cargo sorting by facilitating the selective packaging of microRNAs [35]. Another proposed mechanism suggests a sorting based on uridylation versus adenylation pathway at 3′ end of the microRNAs [36]. Interestingly, it has also been demonstrated that exosomes carry the machinery required for maturation and target identification of microRNAs [37]. Despite the consistent body of articles in the field, the mechanisms behind exosome cargo need to be further elucidated with a promising perspective to engineer them and develop innovative anti-cancer approaches.

## 2. State of the Art: The Role of Exosomes in Cancer Immunomodulation

Exosomes are released in body fluids including urine, saliva, cerebrospinal fluid, breast milk, and blood. Because exosomes are key mediators of intercellular communications between local and distant body districts [38], in a cancerous context they provide a means to sustain tumor growth and aggressiveness. In recent years, literature has reported TEXs and their specific cargo as key regulators of tumor neo-angiogenesis [39,40,41,42], therapy resistance [43,44,45,46], and metastasis pre-niche formation [47,48]. One of the main barriers to neo-cancerous formations devoted to the elimination of damaged and deranged cells is represented by immune system surveillance. Therefore, the complex relationship occurring between the immune system and developing tumors is crucial for cancer development. Tumors establish aberrant communications within the microenvironment and the immune system cells, often resulting in destructive processes, excessive tissue remodeling, loss of tissue architecture, and, ultimately, cancer progression. In details, cancer cells recruit cytotoxic T lymphocytes (CTL) and natural killer (NK) cells and redirect them to facilitate tumor invasion, spreading, and angiogenesis [49,50]. Neoplastic immunomodulation encompasses the expression of specific genes and proteins thereof, which act as important repressors of tumor cell recognition and killing [51,52,53,54,55,56]. In this scenario, TEXs are important mediators of tumor immune escaping. Several tumors, e.g., pancreatic [57], oral cancer [58], head and neck cancer [59], melanoma [60], colorectal carcinoma [61], and gastric cancer [62], regulate T cell homeostasis by releasing TEXs. Nanovesicles released by tumor cells express immunosuppressive molecules such as Fas Ligand (FasL) [63,64], Tumor Necrosis Factor-related apoptosis-inducing ligand (TRAIL), Programmed death-ligand 1 (PD-L1), and Interleukin 10 (IL-10), as well as microenvironment conditioning factors, e.g., transforming growth factor β (TGF-β1), prostaglandin E2 (PGE2), and ectoenzymes engaged in the adenosine pathway (CD39 and CD73) [16,65,66,67].

Liu and colleagues [68] described that TEXs may impair NK function [69,70,71] and interfere with the differentiation of CD14^+^ monocytes into functional dendritic cells (DC) [72]. Zhang and co-authors highlighted a novel immunosuppressive mechanism adopted by myeloid-derived suppressor cells (MDSC) [73]. In particular, activated MDSCs reduce the number of CD4^+^ and CD8^+^ lymphocytes, as well as NK cells, and down-modulate their cytotoxic capabilities as a result of the interaction between exosomal tumor HSP72 (heat shock protein 27), myeloid-derived TLR-2 (Toll-like receptor 2) and MyD88 (myeloid differentiation primary response protein 88) [73].

TEXs have been also been shown to mediate inflammatory processes involving macrophages that are regulators of the host immunity [74,75]. Indeed, during cancer progression, tumor-associated macrophages (TAMs) undergo a conversion from anti- to pro-tumor activity. In line with this evidence, a specific TAM-M2-like cell phenotype secreting high IL-10 and low IL-12 levels suppresses anti-tumor immune response while promoting tissue remodeling and neo-angiogenesis favoring metastatic dissemination [76]. Interestingly, in a recent study it has been demonstrated that, after being primed by TEXs, macrophages display a specific cytokine expression profile, characterized by, among others, IL-4 expression [75]. TEXs have been shown to promote tumor growth by inducing the expansion of Treg and the effector CD4^+^ sub-populations and by fostering their immunosuppressive capacity [77,78,79,80]. Disrupting the aberrant immune system-cancer circuitry may promote an anti-tumor progression effect. A different body of literature supports the notion that, if enforced to express class I and II major histocompatibility complex (MHC) molecules on their surface, TEXs are capable of stimulating an immune reaction against tumors. Along the same lines, TEXs may restore the function of presenting cancer neo-antigens and stimulate immune system reactivity [81,82,83,84,85]. Taken together, this set of evidence suggests an innovative and intriguing potential exploitation of TEXs as a new vaccine and immunization tool for cancer therapy [86].

## 3. Exosomal MicroRNA Cargo Influences the Immune System

**Figure 1:**
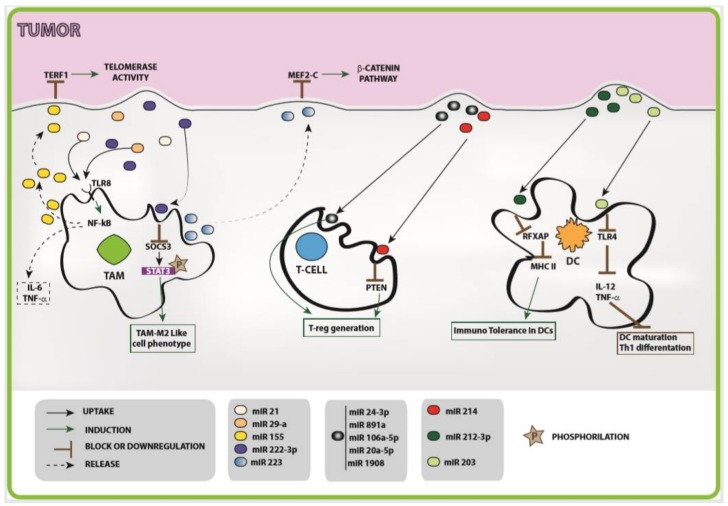
Schematic representation of TEX- and miRNA-mediated crosstalk between cancer cells and the immune system.

It is acknowledged that, when released into “host target” cells, exosomal microRNAs exert their biological function [87,88,89] and, in particular, may reprogram immunity to promote cancer [52]. Thus, shuttled microRNAs represent important messengers for exchanging information between tumor cells, the immune system, and the microenvironment. The crosstalk between cancer cells and their microenvironment is a complex and dynamic molecular network and requires further investigation in order to be completely elucidated. The host immune system is calibrated on discrimination of self and non-self in which both innate and adaptive immune responses are involved. Several groups investigated the role of tumor exosome cargo in altering immune self-tolerance, highlighting its capacity to influence both the innate and the adaptive systems. In Figure 1 the summary of the crosstalk between cancers and immune response is reported.

### 3.1. Innate System Immunomodulation

Macrophages are key actors of host immunity, and TAMs seeded in the tumor microenvironment play a crucial role in inhibiting their immune response [90,91,92]. In recent years, Fabbri and colleagues identified new exosomal microRNA-mediated crosstalk between TAMs and lung [93]. In particular, the authors demonstrated that non-small cell lung cancers (NSCLC) release exosomes containing miR-21 and miR-29a. Upon being intercepted by TAMs, these microRNAs have been shown to bind their specific receptor, i.e., TLR8 (Toll-like receptor 8), thereby activating Nuclear factor kappa-light-chain-enhancer of activated B cells (NF-κB) transcription factor and ultimately leading to an increased secretion of inflammatory cytokines such as IL-6 and Tumor necrosis factor α (TNF-α). The subsequent inflammatory process within the tumor microenvironment supports tumor growth and spreading (Table 1). Along similar lines, another group has recently demonstrated that exosomes produced by epithelial ovarian cancer (EOC) contain miR-222-3p [94]. In this regard, shuttling of such miRNA to macrophages (i) suppressed SOCS3 (cytokine signaling 3) expression; (ii) favored consequent phosphorylation of STAT3 (Signal transducer and activator of transcription; and (iii) caused polarization of macrophages towards a M2 TAM-like phenotype. As a result, an induced TAM phenotype promotes the progression of epithelial ovarian cancer [94] (Table 1). A further recent study showed that macrophages can release exosomes carrying miR-223, which down-modulates the MEF2C (myocyte-specific enhancer factor 2C)-β-catenin pathway and enhances the invasiveness of breast cancer cells [95] (Table 1). An intriguing, diverse mechanism of miRNA-mediated exchange of molecular information was recently described in neuroblastoma by Challagundla and co-authors [96]. Reportedly, TEXs transfer miR-21 to TAMs, which activate TLR8 receptor and up-regulate the expression of miR-155. The latter is an oncomiR that is frequently overexpressed in cancer and is transcriptionally regulated by the NF-κB pathway. Subsequently, TAM-derived exosomes, enriched for miR-155, travel back to the tumor, and, upon uptake by cancer cells, miR-155 binds to TERF1 (Telomeric Repeat Binding Factor 1) mRNA. The resulting activation of telomerase activity ultimately accounts for resistance of neuroblastoma to Cisplatin treatment (Table 1). These aforementioned results represent the first evidence of miRNA-mediated exosomal shuttling of paracrine, immunomodulatory signals associated with cancer progression. Finally, the reported data underpin the sophisticated and complex crosstalk between cancer cells, their microenvironment, and the immune system. 

### 3.2. Adaptive System Immunomodulation

The anti-tumor response encompasses the activation of specific cells among the immune naïve repertoire, e.g., dendritic cells (DCs), natural killer cells (NK cells), and CD8^+^ effector T cells. Such a “swiss-army knife” constitutes a natural body defense against cell senescence and DNA damage. However, during their development neoplastic cells disrupt the host’s natural defense system and evade the immune surveillance. Indeed, tumor cells escape from the control by immune system via “re-programming” of its component cells and of the surrounding microenvironment.

Bell and Taylor reported that proteins, as well as specific miRNA (miR-24-3p, miR-891a, miR-106a-5p, miR-20a-5p, and miR-1908), shuttled by exosomes could modulate T-cell function in nasopharyngeal carcinoma (NPC) [5,99] (Table 1). In another study, TEX-derived miR-214 down-regulates PTEN (phosphatase and tensin homolog) levels and promotes the expansion of the Treg sub-effector population in a murine model (Table 1). In this scenario, Treg cells activated by miR-214 produce high levels of IL-10, thereby promoting immunosuppression and facilitating tumor engraftment in mice [100]. Along similar lines, Okoye and colleagues showed that exosomes derived from Treg cells modulate PTGS2 (prostaglandin-endoperoxide synthase 2) activity by shuttling PTGS2-specific microRNAs. Silencing of PTGS2 results in the inhibition of Th1 effector responses [5,105]. Furthermore, a group of exosomal miRNAs secreted by pancreatic cancer cells may mediate immunomodulation in dendritic cells [101]. In particular, exosomal miR-212-3p inhibits the expression of RFXAP (regulatory factor X-associated protein), a key transcription factor for the *MHC-II* gene, with a consequent decrease in class II MHC expression and induction of immune tolerance in DCs [101] (Table 1). In an additional pancreatic adenocarcinoma model, it has been shown that TEX-derived miR-203 down-regulates TRL4 expression while affecting TNF-α and IL-12 production and impairing DC maturation and Th1 differentiation [89] (Table 1). Finally, in vitro and in vivo experiments demonstrated that two different microRNAs released from DC exosomes, i.e., miR-146a and miR-155, are involved in the inflammation process and regulate cellular responses to endotoxin [97,98] (Table 1).

## 4. Exosomal MiRNAs and Hypoxia

During their growth, tumors are repeatedly challenged by low oxygen tension (hypoxia) and cellular adaptation. Cancer-induced micro-environmental conditions ultimately resulting in neoplasia survival and resistance to therapy. Moreover, hypoxia has been recently shown to play a key role in cancer immunosuppression, since it impairs the cytolytic activity of CTLs and NKs [106,107]. Similarly, tumor hypoxia enhances the release of exosomes by breast cancer cells and causes the excretion of qualitatively different vesicles that are able to block NK cell function [98,102]. Berchem and colleagues demonstrated that the immunosuppressive effect of miRNAs is orchestrated in conjunction with TGF-β, whereby both TGF-β and miRNAs are shuttled by exosomes and cooperate in tumor immunoescape. Whilst TGF-β decreases NK cell cytotoxicity by affecting the levels of NKG2D, miRNAs reduce CD107a expression [102] (Table 1). Also, hypoxic TEXs contain elevated levels of miR-210 and miR-23a, in line with previous evidences correlating miR-210 with tumor hypoxia and immune escape [103]. Similarly, exosomes released by hypoxic melanoma cells express miR-4498, which regulates CD83, an immunostimulatory molecule that is critical for T cell activation. Mechanistically, TEX-derived miR-4498 decreases CD83 levels, thereby impairing immune system responses against melanoma cells [102,104] (Table 1).

## 5. Exosome Mediated Therapy and Immunomodulation

The capability of exosomes to shuttle diverse types of molecules has fostered several efforts to design and improve methods for their engineering. Recent studies proposed virus—as well as non-virus-mediated—approaches for exogenous exosomal loading and antitumor therapeutic delivery. In addition, a large body of research has focused on discovery of exosomal surface antigens, with the aim of improving and directing exosome uptake. A comprehensive review of the topic, i.e., modification of exosome cargo for cancer therapy, is available in Gilligan and Dwyer [108].

Intriguingly, exosomes could serve as a functional immunization reagent for the production of a new generation of vaccines. Alternatively, autologous nanovesicles could be engineered to counteract tumor-induced immune suppression. As an example, hMUC1 (human Mucin1)-modified exosomes have been used for stimulating an autologous and allogenic immune response in vivo [109]. Along the same lines, pre-immunization with TEXs containing RAB27a (Ras-related protein) has been shown to cause significant inhibition of tumor growth in vivo [110].

In the last few years, human TRAIL-engineered exosomes were used to induce apoptosis in melanoma and lymphoma cell lines [111,112]. Furthermore, exosomes loaded with IL-18 and IL-2 were used to stimulate the immune system against tumors [86,113].

Recently, exosomes have gained increasing interest as an engineerable tool for transporting therapeutic microRNAs [108]. Notably, microRNA, proteins, or drugs loaded on exosomes have been delivered to tumor cells and across the blood brain barrier (BBB) [114]; for example, miR-134 was proven to be down-regulated in exosomes isolated by breast tumors, thus controlling HSP90 (heat shock protein 90), level and acting as a tumor suppressor. In particular, miR-134 overexpressing exosomes were delivered against triple-negative breast cancer cells and resulted in a significant reduction of STAT5B, HSP90 and Bcl-2 levels, with a concomitant impairment of cellular viability and enhancement of cisplatin-sensitivity [115]. Furthermore, Munoz and colleagues showed that miR-9 is up-regulated in glioblastoma multiforme (GBM) cells and is associated with resistance to temozolomide (TMZ) [116]. Since miR-9 controls the expression of P-glycoprotein, a drug efflux transporter, the authors sought to engineer mesenchymal stem cells (MSCs) with anti-miR-9. It is known that MSCs migrate to the sites of cancers and produce high levels of vesicles. Therefore, mainly through the release of vesicles containing anti-miR-9, MSCs were able to sensitize GBM cells to TMZ [116].

In another study, adipose tissue-derived MSCs (AMSCs) were engineered with miR-122 [117]. Hepatocellular carcinoma (HCC) patients with low levels of miR-122 are likely to bear metastases and display poor prognosis. Therefore, exosomes isolated from AMSCs overexpressing miR-122 were used to challenge tumor cells, resulting in significant reduction of proliferation, invasiveness, and increased sensitivity to chemotherapy and sorafenib treatment both in vitro and in vivo [117]. Altogether, these cited studies highlight the intriguing opportunity to exploit engineered exosomes as therapeutic tools. Finally, we reported a selection of studies pointing to the potential clinical relevance of exosome-based tumor vaccines and to novel and improved immunotherapeutic approaches against cancer.

## 6. Discussion

TEXs represent an intriguing new field of investigation for the discovery of sophisticated cell interactions. Moreover, molecular analysis of TEXs is at the forefront of providing the scientific community with a promising, novel and improved means for cancer diagnosis and therapeutics. Here we reported several examples of evidence of the role of exosomes in induction of tumor immune tolerance. Since exosomes are largely abundant in biological fluids and this holds particularly true in cancer settings, they have recently received great attention as key vehicles of information within liquid biopsies. Indeed, analysis of exosome content represents a promising tool for discovering new biomarkers of early cancer insurgence. On the one hand, characterization of TEXs offers a reliable, sensitive, non-invasive monitoring method starting from minute amounts of biological samples. As a proof of principle, several cancer-specific markers have been recently identified in exosomes. Examples include but are not limited to (i) CD-24 and EpCAM, which were described to be over- and under-represented, respectively, in isolated exosomes from breast cancer sera [118]; (ii) Glypican-1, a cell-surface proteoglycan reported as up-regulated in serum exosomes of patients with pancreatic cancer [119]; (iii) Survivin, whose levels were found increased in exosomes derived from prostate cancer when compared to those isolated from healthy donors [120]; and (iv) TGF-β1 and MAGE 3/6 (Melanoma associated Antigen 3/6), shuttled from exosomes isolated from ovarian cancer patients [121]. On the other hand, we reviewed the literature by focusing our attention on microRNA-specific exosomal cargo. In particular, we concentrated on the ability of exosomal microRNA to favor tumor aggressiveness and metastatic spreading by both inhibiting immune response and aberrantly activating immune system cells in a pro-tumorigenic fashion. This is a hotly debated scientific topic which provides an intriguing perspective with important implications in cancer immunotherapy.

Recently, particular attention has been devoted to exosomal miRNA cargo. As a matter of fact, microRNAs are crucial regulators of tumorigenesis and represent a new frontier in cancer diagnosis and prognosis, as well as in the prediction of therapeutic responsiveness. In this regard, recent evidence has proven the usefulness of miRNAs in disease progression monitoring [122,123]. Examples of miRNAs that were implicated in TEX-mediated functions include, but are not limited to, (i) miR-21, miR-141, miR-200a, miR-200c, miR-200b, miR-203, miR-205, and miR-214, all of which were found to be expressed in circulating TEXs isolated from ovarian cancer patients [124]; and (ii) miR-21 and miR-1246, identified as diagnostic and prognostic markers in exosomes from esophageal squamous cell cancer (ESCC) [125,126,127].

Intrinsic, as well as acquired, drug resistance represents one of the major hurdles in the treatment of advanced cancers. Since persistent, drug-resistant tumor cells release a large quantity of exosomes, their microRNA repertoire may actively participate in shaping therapeutic responsiveness. Examples include, but are not limited to, (i) miR-125b, which increases the sensitivity of breast cancer patients to anthracycline-based chemotherapy [128]; (ii) miR-122, which confers taxol resistance via upregulation of Septin-9 in liver cancer [129]; (iii) miR-9, which modulates BRCA1 (Breast Cancer Type 1 susceptibility protein) and the DNA damage repair capacity of epithelial ovarian cancer cells, ultimately determining their chemosensitivity [130]; and (iv) IncARSR (lncRNA Activated in RCC with Sunitinib Resistance), whose exosome-mediated transmission confers Sunitinib resistance in a model of renal cell carcinoma [131,132].

Taken together, the data reported here stress the crucial role of exosomes and their microRNA cargo in the future perspectives of modern oncology. In recent years, several research groups have devoted their efforts towards studying exosomes and, in particular, exosomal miRNAs as new diagnostic and prognostic tools. Furthermore, stimulated by the encouraging results obtained in the field, the scientific community is envisioning new methods of therapeutic administration, as well as vaccines, based on exosomes. The investigation of TEXs provides us with new information to fill important gaps of knowledge in tumor heterogeneity and its interaction with the microenvironment and the immune system.

## Figures and Tables

**Table 1:** Role of miRNAs in immunomodulation and cancer.

microRNA	Tumor	Action	References
miR-21	NSCLC; Neuroblastoma	Increase inflammation	[93,96]
miR-29a	NSCLC	Increase inflammation	[93]
miR-222-3p	Epithelial ovarian carcinoma	M2 TAM-like macrophages polarization	[94]
miR-223	Breast Cancer	Invasiveness enhancement	[95]
miR-155	Neuroblastoma; Dendritic cell	Telomerase activity alteration and Cisplatin resistance; Increase inflammation	[96,97,98]
miR-24-3p	Nasopharyngeal carcinoma	Anti-tumor immunity suppression	[4,99]
miR-891a	Nasopharyngeal carcinoma	Anti-tumor immunity suppression	[4,99]
miR-106a-5p	Nasopharyngeal carcinoma	Anti-tumor immunity suppression	[4,99]
miR-1908	Nasopharyngeal carcinoma	Anti-tumor immunity suppression	[4,99]
miR-20a-5p	Nasopharyngeal carcinoma	Anti-tumor immunity suppression	[4,99]
miR-214	Sarcoma	Anti-tumor immunity suppression	[100]
miR-212-3p	Pancreatic cancer	Immuno tolerance induction	[101]
miR-203	Pancreatic adenocarcinoma	Anti-tumor immunity suppression	[89]
miR-146a	Dendritic cell	Reduce inflammation	[97,98]
miR-210	Lung cancer; myelogenous leukemia cell lines	Immune escaping	[102,103]
miR-23a	Lung cancer; myelogenous leukemia	Immune escaping	[102]
miR-4498	Melanoma	Anti-tumor immunity suppression	[104]
